# Differentiation of Crohn’s Disease-Associated Isolates from Other Pathogenic *Escherichia coli* by Fimbrial Adhesion under Shear Force

**DOI:** 10.3390/biology5020014

**Published:** 2016-04-01

**Authors:** Sabine Szunerits, Oleksandr Zagorodko, Virginie Cogez, Tetiana Dumych, Thibaut Chalopin, Dimitri Alvarez Dorta, Adeline Sivignon, Nicolas Barnich, Anne Harduin-Lepers, Iban Larroulet, Aritz Yanguas Serrano, Aloysius Siriwardena, Amaia Pesquera, Amaia Zurutuza, Sébastien G. Gouin, Rabah Boukherroub, Julie Bouckaert

**Affiliations:** 1 Institute of Electronics, Microelectronics and Nanotechnology (IEMN), UMR 8520 CNRS, Université Lille 1, Avenue Poincaré-BP 60069, 59652 Villeneuve d’Ascq, France; morjakzzz@gmail.com (O.Z.); rabah.boukherroub@iemn.univ-lille1.fr (R.B.); 2 Unité de Glycobiologie Structurale et Fonctionnelle (UGSF), UMR 8576 CNRS, Université de Lille, 59000 Lille, France; virginie.cogez@univ-lille1.fr (V.C.); tetiana.dumych@gmail.com (T.D.); anne.harduin@univ-lille1.fr (A.H.-L.); 3 Department of Cytology, Histology, Embryology, Danylo Halytsky Lviv National Medical University, Pekarska Str 69, 79010 Lviv, Ukraine; 4 Chimie Et Interdisciplinarité, Synthèse, Analyse, Modélisation (CEISAM), UMR 6230 CNRS, LUNAM Université, 44322 Nantes Cedex 3, France; thibaut.chalopin@univ-nantes.fr (T.C.); dimitri.alvarez-dorta@univ-nantes.fr (D.A.D.); sebastien.gouin@univ-nantes.fr (S.G.G.); 5 M2iSH, Microbes, Intestin, Inflammation and Susceptibility of the Host , UMR1071 Inserm/Université d’Auvergne, USC INRA 2018, 63000 Clermont-Ferrand, France; adeline.sivignon@udamail.fr (A.S.); nicolas.barnich@udamail.fr (N.B.); 6 SENSIA SL, Poligono Aranguren, 9, Apdo, Correos 171, 20180 Oiartzun, Spain; ilarroulet@seimcc.com (I.L.); aritz_yanguas@hotmail.com (A.Y.S.); 7 Glycochimie des Antimicrobiens et des Agroressources, UMR 7378 CNRS, Université de Picardie Jules Verne, 80000 Amiens, France; aloysius.siriwardena@u-picardie.fr; 8 Graphenea S.A., Tolosa Hiribidea, 76, E-20018 Donostia, Spain; a.pesquera@graphenea.com (A.P.); a.zurutuza@graphenea.com (A.Z.)

**Keywords:** adherent-invasive *Escherichia coli*, shear force, surface plasmon resonance, graphene, heptyl α-d-mannose

## Abstract

Shear force exerted on uropathogenic *Escherichia coli* adhering to surfaces makes type-1 fimbriae stretch out like springs to catch on to mannosidic receptors. This mechanism is initiated by a disruption of the quaternary interactions between the lectin and the pilin of the two-domain FimH adhesin and transduces allosterically to the mannose-binding pocket of FimH to increase its affinity. Mannose-specific adhesion of 14 *E. coli* pathovars was measured under flow, using surface plasmon resonance detection on functionalized graphene-coated gold interfaces. Increasing the shear had important differential consequences on bacterial adhesion. Adherent-invasive *E. coli*, isolated from the feces and biopsies of Crohn’s disease patients, consistently changed their adhesion behavior less under shear and displayed lower SPR signals, compared to *E. coli* opportunistically infecting the urinary tract, intestines or loci of knee and hip prostheses. We exemplified this further with the extreme behaviors of the reference strains UTI89 and LF82. Whereas their FimA major pilins have identical sequences, FimH of LF82 *E. coli* is marked by the Thr158Pro mutation. Positioned in the inter-domain region known to carry hot spots of mutations in *E. coli* pathotypes, residue 158 is indicated to play a structural role in the allosteric regulation of type-1 fimbriae-mediated bacterial adhesion.

## 1. Introduction

The Gram-negative *Escherichia coli (E. coli)* species express type-1 pili or fimbriae, which are organelles integral to host receptor recognition and attachment [1]. A mannose-recognizing FimH adhesin is found at the tip of the type-1 pilus [2]. The adhesin FimH is a two-domain adhesin (TDA) with an *N*-terminal mannose-binding lectin domain connected by a flexible linker to a pilin anchoring it to FimG and FimF in the tip fibrillum. Natural variations in amino acid composition were found to regulate the pathoadaptivity of *E. coli* [3], not frequently in or near the conserved mannose-binding pocket, but predominantly at the interface between the lectin and pilin domains of the FimH TDA. The structural basis for these observations remained obscure, until the crystal structure of the whole tip of *E. coli* type-1 fimbriae (a FimH-FimG-FimF-FimF’-FimC macromolecular assembly, PDB Entry Code 3JWN [4]) from the *E. coli* F18 fecal strain revealed that the FimH lectin is in a different conformational state when not bound to mannosidic receptors. Extensive quaternary contacts between the lectin and the pilin domains of the FimH TDA keep the lectin domain in a compressed conformer with low affinity for its receptors [5]. This conformation in the fimbrial tip assembly thus significantly differs from the extended, high-affinity conformer of the FimH lectin domain, as found in the crystal structures of the soluble lectin [6] or of the full-length FimH TDA bound onto its steric chaperone FimC [7,8].

Here, we demonstrate the importance of amino acid variations in FimH on the capacity of the whole FimH TDA to extend its structure, adopt the high-affinity conformation and initiate fimbrial stretching and further uncoiling [9]. Early electron microscopic analysis already pointed to the importance of the incorporation of FimH into *E. coli* type-1 fimbriae to the extensibility of the stubby and flexible tip fibrillum [2]. Thomas and co-workers observed that the adhesion of *E. coli* to mannose-coated surfaces in laminar flow chambers is enhanced by shear stress [10]. These authors attributed this phenomenon to the formation of force-enhanced allosterically regulated catch bonds between the type-1 fimbrial adhesin and surface-attached mannosidic moieties [11]. The FimH TDA opens up around the linker region, through rupture of the inter-domain interactions under the influence of shear stress [12]. The domain separation liberates the FimH lectin that expands to make high-affinity catch bonds to a mannosidic glycan receptor [5]. Evidence for this model was further strengthened by data from experiments using molecular force spectroscopy [13], structural dynamic simulations [9,14,15] and kinetic measurements [15]. Despite the coincidence of the inter-domain region of the FimH TDA with hot spots of variation in *E. coli* pathotypes [16], no structural role has hitherto been attributed to naturally-occurring variant amino acids in the allosteric regulation of FimH affinity when *E. coli* experience shear stress. Therefore, we decided to examine 14 *E. coli* clinical isolates, from a variety of niches in the human host, including the urinary tract, the intestine and loci of infections in knee and hip prostheses, for their capability to adhere under increasing shear stress.

Shear is the dominant effect of fluid flow and can be controlled by changing the flow rate through microfluidic channels. The drag force exerted per surface area is called shear or shear stress (τ). Fimbriated bacteria can relieve some of the shear stress by spreading their fimbrial organelles and pulling themselves closer to the plasmonic interface [17]. When exceeding an optimal flow rate, the fimbrial bonds break, and the bacteria detach. Utilizing flow cells, the influence of several parameters, such as pH, ionic strength and surface chemistry, on bacterial adhesion has been investigated [18,19,20]. The shear force threshold varies from strain to strain and depends moreover on the specific chemical composition of the substrate surface under investigation. Different receptors of FimH, such as yeast mannan, bovine serum albumin-linked mannose and guinea pig red blood cells, have previously been used to test their influence on shear-force-enhanced adhesion of *E. coli* [13,21].

It was recently demonstrated that the growth of *E. coli* on Au interfaces modified with carbohydrates can be followed with very high sensitivity using surface plasmon resonance (SPR) detection [22]. We have shown that the adhesion of uropathogenic *E. coli* UTI89 can be conveniently studied by following the SPR signals on the graphene-coated plasmonic interfaces [23,24]. Graphene has been actively pursued as a versatile platform for biomedical-related applications [25], and graphene-coated gold interfaces have been shown to be excellent tools for the measurement of carbohydrate-lectin affinities [26], for the screening of DNA-DNA [27] and for the aptamer-lysozyme [28] interactions, as well as for promoting the adhesion, proliferation and differentiation of various cells [29,30,31].

The great biocompatibility of graphene-based plasmonic interfaces and the ease of their chemical modification with heptyl α-d-mannopyranoside (HM) are taken advantage of here [23] to study the influence of flow rate on shear force-enhanced bacterial adhesion. This flow cell experimental set-up with SPR detection was applied to 14 *E. coli* pathovars for the comparison of their adhesion under quasi-identical experimental conditions. Remarkably, a consistently lower threshold to withstand shear stress was observed for adherent-invasive *E. coli* (AIEC) isolates from Crohn’s disease patients compared to other *E. coli* pathotypes. The most affected AIEC strains contain mutations in the inter-domain region of the FimH TDA (Table 1) [32], which coincides with the region where *E. coli* pathovars differ [3]. Our data confirm an in vitro model that closely mimics the FimH interaction with the glycocalyx of the host cells, to study mannose-specific adhesion of *E. coli* pathovars [3].

## 2. Materials and Methods

### 2.1. Functionalization of Au-Graphene SPR Interfaces with Glycans

Au-graphene biosensors were prepared as described in the Supplementary Materials (Figure S1). Aminoheptyl α-d-mannopyranoside (HM) [33], nitrophenyl α-d-mannopyranoside (3), nitrophenyl α-d-glucopyranoside (4) and nitrophenyl β-d-glucopyranoside (5) were synthesized as reported [34]. The Au-graphene interfaces were functionalized through simple immersion for 2 h into the respective carbohydrate solution (500 µM). Subsequently, the interfaces were rinsed twice with deionized water and dried under a gentle stream of nitrogen. XPS experiments were performed for quality control of the interfaces, and the amount of carbohydrates loaded on the Au-graphene interface was calculated from these measurements and verified by treatment with a phenol/H_2_SO_4_ solution [35] (Table S1).

### 2.2. Selection and Growth of Escherichia coli Pathovars

Each of the *E. coli* strains (Table 1) has been inoculated on Luria broth (LB)-agar, from which a few colonies were selected for an overnight preculture in liquid LB. Upon a 100-fold dilution in liquid LB in a flask with a large air-liquid interface, bacterial culture was continued under static conditions at 37 °C during 48 h to optimize type-1 fimbrial expression. The bacterial pellet was obtained upon centrifugation, washed three times in ice-cold phosphate-buffered saline (PBS) and resolubilized in PBS to an optical density at 600 nm (OD_600nm_) of around 10 as the starting concentration for hemagglutination. A spectrophotometrically-obtained optical density measured at 600 nm of 1.0 conforms to on average 0.66 × 10^9^ colony-forming units (cfu)·mL^−1^. The same bacterial preparations were used for hemagglutination and SPR experiments. Each *E. coli* strain was cultured at least twice under these same conditions.

### 2.3. Bacterial Hemagglutination

Guinea pig erythrocytes (Eurogentec, Liège, Belgium) were agglutinated by the different *E. coli* strains. The *E. coli* strains, with a starting concentration as determined by spectrophotometry, were two-fold diluted in PBS, and 50 µL of each dilution were deposited in round-bottomed 96-well plates. The red blood cells were washed twice in the ice-cold PBS, diluted to 5% of the original solution, and 50 µL were added to the wells. The plates were left on ice for one night before read-out of the hemagglutination titer.

### 2.4. SPR Measurements at Different Flow Rates

The SPR measurements were performed with an Indicator-G instrument (SENSIA, San Sebastian, Spain) equipped with a laser light of a 650-nm wavelength and two channels that are each served by 60-µL loops. The channels are 12-mm long and have an inner diameter of 0.5 mm, which allow laminar flow (Re = 3) at speeds from 4 to 100 µL·min^−1^. The bacterial strain solutions were diluted to 1 × 10^8^ cfu/mL in the running buffer PBS and injected at different flow rates, 5, 10, 20, 30, 50 and 100 µL·min^−^^1^, corresponding to 5, 10, 30 and 100 mPa, respectively, into the two flow channels. At the 30-min time point after injection of bacteria, the bacterial load on the surface was stable for all flow rates (Figure S2), and the SPR intensities (pixels) were read out. Then, the injection was stopped, and after a short wash in PBS, the surface was regenerated using 50 mM NaOH to interrupt the FimH-HM interactions. The standard deviations on the average of two measurements per particular flow rate, using at least two independent preparations of bacteria, are shown by error indicators on top of the column bars.

### 2.5. fimH Sequencing of Novel Bacterial Clones

The *fimH* gene was amplified in PCR reactions using colonies as the template. The primers used to amplify fimH were uti8+4913338 and uti8-4914820 [36] (Uti8+4913338 ACCGCGCAAAACATCCAGTT, Uti8-4914820 GGTATTCGGCATTGGCCTGA). Amplification was achieved with Phusion High-Fidelity DNA polymerase (Thermo Scientific, Villebon-sur-Yvette, France) according to the manufacturer’s instructions. Reactions were held at 98 °C for 1 min, cycled 35 times between 98 °C (30 s), 63 °C (30 s) and 72 °C (1 min 30 s) and finished at 72 °C for 5 min. Products were analyzed by electrophoresis on agarose gel, and bands were cut and purified (NucleoSpin^®^ Gel and PCR Clean up, Macherey-Nagel, Düren, Germany), then submitted for Sanger sequencing by GATC Biotech, Konstanz, Germany. Sequencing primers were fimhZLfp ATGAAACGAGTTATTACCCTGT, fimhZLrp TTGATAAACAAAAGTCACGCCAAT, Uti8+4913927 CCGGTGGCGCTTTATTTGAC and Uti8+4913338. Sequence comparisons were done using the BLASTN 2.3.1+ program at NCBI [37].

### 2.6. Structural Analyses

The overlays and comparisons of crystal structures were performed using the graphics program Coot [38] and Pymol [39]. Crystal structures obtained from the PDB (4BUQ, 1KLF) were a FimH variant from *E. coli* J96 that features an identical amino acid sequence as that of the K12 strain (V27, N70 and S78 residues; see Table 1). The crystal structure of the type-1 fimbrial tip (3JWN) incorporates the FimH variant of fecal *E. coli* F18 (A27, S70 and N78 residues).

## 3. Results

### 3.1. Mannose-Specific Adhesion of E. coli UTI89 to Graphene-on-Gold Surfaces under Flow

**Figure 1:**
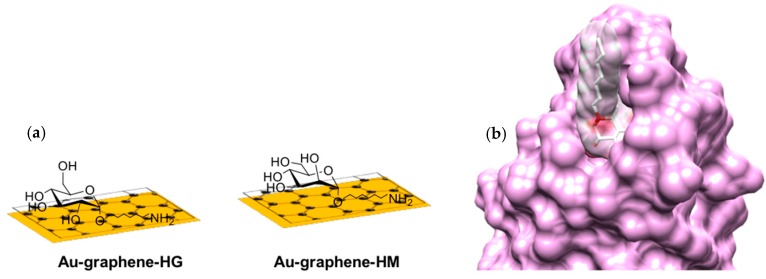

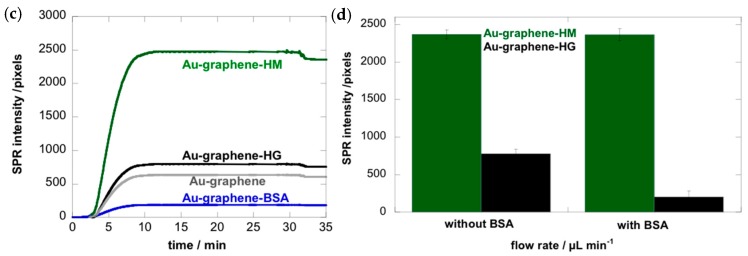
SPR detection of *E. coli* UTI89 binding. (**a**) Au-graphene-HM (green) and Au-graphene-HG (black) carbohydrate-modified interfaces; (**b**) van der Waals surfaces of the FimH-HM complex as observed in the crystal structures (PDB Entry 4BUQ); (**c**) sensorgrams of 10^8^ cfu·mL^−1^ bacterial concentrations at a flow rate of 30 µL·min^−1^ on those interfaces and control interfaces Au-graphene (grey) and Au-graphene-BSA (blue). The read-out of the SPR intensities was made after 30 min of flow. (**d**) Coating with BSA of Au-graphene-HM and Au-graphene-HG interfaces reduced the non-specific interaction of *E. coli* with the interface.

**Figure 2:**
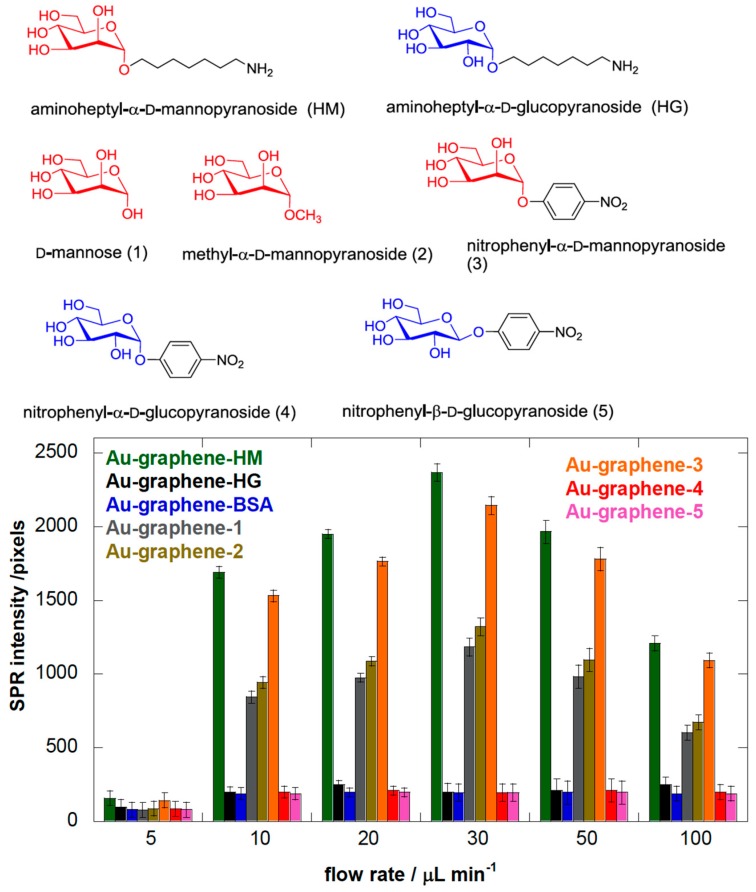
The chemical structures of carbohydrates used to modify the Au-graphene interface for assessing interaction with *E. coli* UTI89. The bar diagram represents the SPR intensities (in pixels) under different flow rates, upon the addition of *E. coli* (10^8^ cfu·mL^−1^) to BSA-coated Au-graphene interfaces modified with the different carbohydrate structures. The background level of *E. coli* sticking non-specifically to the interfaces was determined at around 200 pixels based on its behavior on BSA- and glucose-modified Au-graphene.

**Figure 3:**
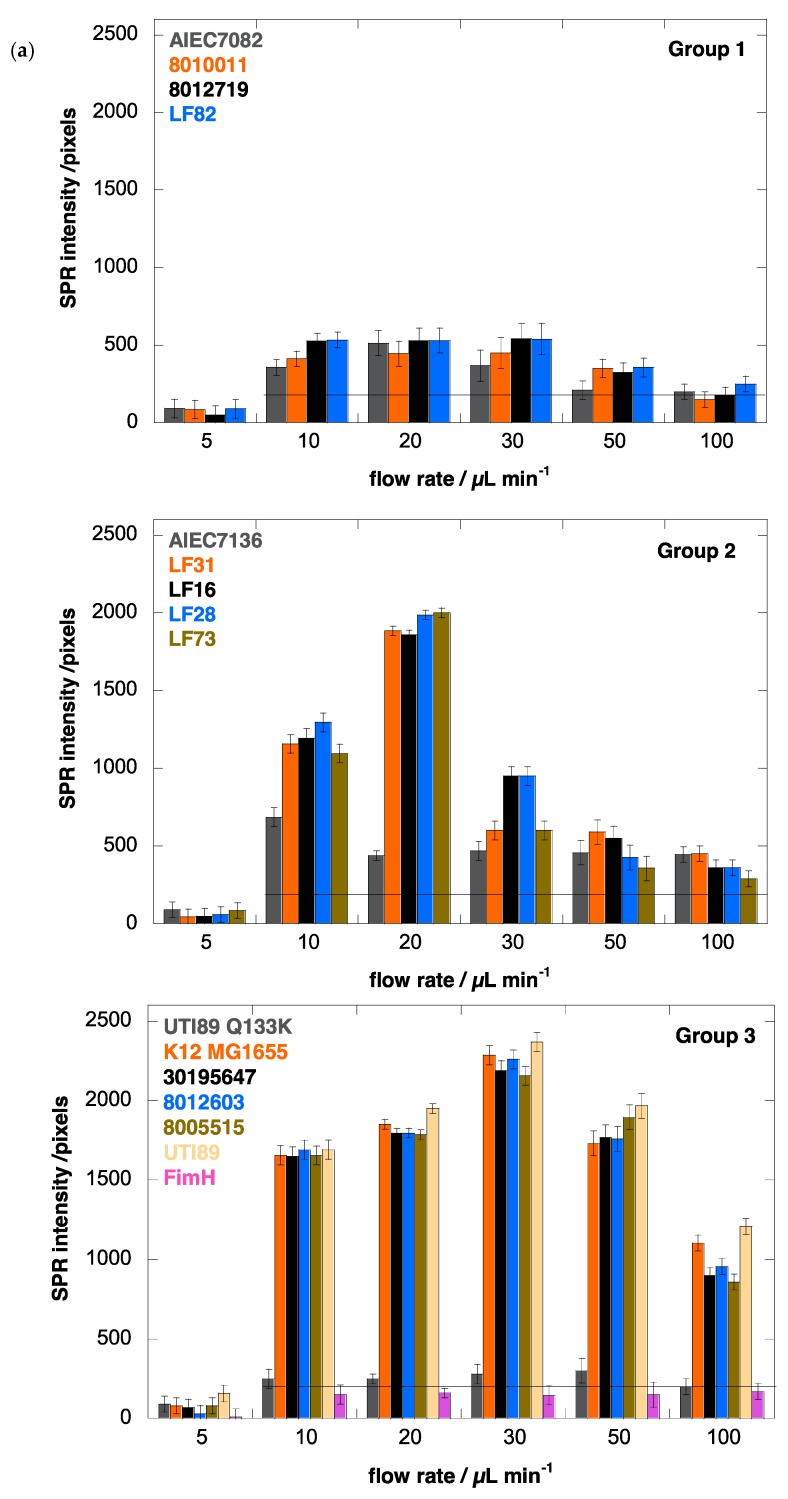

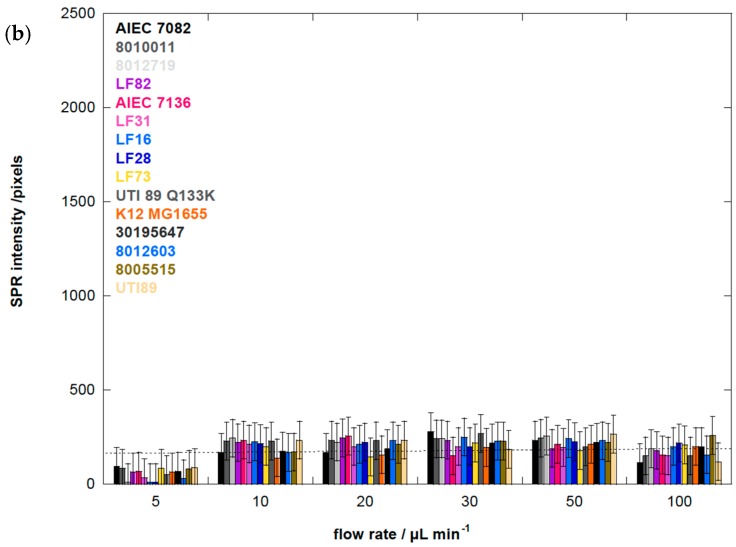
(**a**) Binding affinity of different *E. coli* strains, at 10^8^ cfu/mL, to aminoheptyl α-d-mannopyranoside (**1**) modified Au-graphene as a function of flow rate: Group 1 is *quasi* indifferent to flow rate; Group 2 has a low dependence on shear force with an optimal adhesion near 20 μL·min^−1^. Adhesion of Group 3 *E. coli* is strongly enhanced by shear, with the highest SPR signal above 2000 pixels and at an optimal flow rate of 30 μL·min^−1^. As a control, the *E. coli* UTI89 bearing the FimH Q133K mutant, disabled for mannose binding, indicates the background level for non-mannose-specific bacterial sticking at 200 pixels. Purified FimH lectin domain (FimH, residues 1–158) at a concentration of 1 μM was used as an evaluation of the sensitivity of detection close to the plasmonic surface, and its binding is, as expected, shear-force independent. (**b**) Binding affinity of different *E. coli* strains, at 10^8^ cfu/mL, to aminoheptyl α-d-glucopyranoside (**2**) modified Au-graphene as a function of flow rate. The broken line indicates 200 pixels of SPR intensity, which is estimated as background noise.

The interaction of *E. coli* with host epithelial receptors is mediated by the FimH two-domain adhesin (TDAs), positioned at the tip of multimeric hair-like appendages, called type-1 fimbriae [1,40]. Although mannose is a good receptor for FimH displaying micromolar affinities [6], synthetic monovalent mannosides bearing aromatic [41] or simple alkyl [6] aglycons in the α-anomeric position exhibit nanomolar binding affinities for FimH. The interaction between *E. coli* (at a concentration of 10^8^ cfu·mL^−1^) and carbohydrate-coated Au-graphene interfaces (Figure S1) was investigated in real time using label-free interaction analysis in an SPR assay (Figure 1). The amino-armed analogue of heptyl α-d-mannoside (HM) was identified in this work as a prototype ligand for the modification of Au-graphene, because the presence of the amino end group allows for a tight control of carbohydrate loading by X-ray photon spectroscopy (XPS) analysis (Table S1). It also permits a direct comparison with earlier measurements of *E. coli* on gold sensor chips, where the amino group on HM was used for the covalent immobilization of the ligand [23]. Post-incubation of the mannoside-modified Au-graphene SPR surface in bovine serum albumin (BSA) solutions helped to strongly decrease the non-specific interaction between *E. coli* and the Au-graphene surface (Figure 1d). Non-specific protein-mediated interactions with the graphene are due to the strong interaction of protein with the rich π-conjugation structure of graphene [42]. Post-coating with BSA also helps to significantly reduce the aspecific binding to the Au-graphene-HG surfaces that were prepared with the notion that the FimH adhesin from *E. coli* UTI89 cannot specifically interact with glucosides [6] (Figure 1, Figure 2 and Figure 3b). The Au-graphene surfaces were immersed with BSA prior to the recording of further SPR experiments (Figure 2 and Figure 3).

### 3.2. Heptyl α-d-Mannose Integrates into Graphene in a Way that Is Very Favorable for the Specific Detection of the FimH Adhesin from E. coli

We investigated mannosides bearing a nitrophenyl group in the anomeric position to favor aromatic interactions with the graphene, because this approach is a commonly used strategy for the integration of ligands [43,44,45]. Nitrophenyl α-d-glucoside (4) and the corresponding β-anomer (5) were used as negative controls. XPS analyses indicated that Au-graphene is less loaded with mannose (1) and methyl α-d-mannoside (2) than with aminoheptyl α-d-mannoside (HM) and nitrophenyl α-d-glucosides (4), in agreement with the degree of carbohydrate loading quantified by using an optimized Molisch test [35] (Table S1). The SPR results showed that Au-graphene modified with ligand (HM) exhibited the strongest affinity for *E. coli* UTI89 (Figure 2). A gradual decrease of the binding of *E. coli* was observed, with aminoheptyl α-d-mannoside > nitrophenyl α-d-mannoside >> nitrophenyl α-d-glucoside ≈ nitrophenyl β-d-glucoside, in accordance with the known binding preferences of the FimH adhesin [6]. Mannose- (1) and methyl α-d-mannoside- (2) modified interfaces displayed largely decreased SPR responses when compared to the aminoheptyl and nitrophenyl analogues. This might have partially resulted from the lower carbohydrate loading for the latter (Table S1), although this is more probably due to their much lower affinity for FimH. More importantly, it is known from the crystal structures of FimH that the entire mannosaccharide, except for its α-anomeric hydroxyl group, is buried into the FimH pocket [8]. Its presence on graphene and binding by FimH seem to be incompatible events, and *E. coli* adhesion on mannose-modified graphene indeed exhibits rather has a transient character [24]. This is in contrast with further experiments where Au-graphene interfaces modified with the HM ligand exhibited a very stable bacterial adhesion under a variety of different flow rates (Figure 2).

### 3.3. Adhesion of E. coli UTI89 to Mannosylated Surfaces Is Enhanced under Shear Flow

We were intrigued how varying the flow rate would result in a change in the binding capacity of *E. coli*. The microfluidic channel of the Indicator-G instrument allowed varying the flow rate between 5 and 100 µL·min^−1^, corresponding to shear stresses between ≈ 5 and 100 mPa (0.005–0.1 pN/μm^2^), a range used by others [11,19]. Figure 2 shows the binding of *E. coli* UTI89 at six different flow rates. An increase in flow rate will in the first instance stimulate encounters of the microbes with the substrate due to an increase in microbial transport [46,47]. This effect was observed for all bacterial strains above 5 µL·min^−1^ (Figure 3). *E. coli* adhesion to Au-graphene-HM interfaces was increased more than 15 times upon the change of the flow rate from 5 to 30 µL·min^−1^ (Figure 2). The high SPR signals on Au-graphene-HM interfaces indicate a carbohydrate-specific induction mechanism underpinning the flow rate-dependent adhesion of *E. coli* to this surface. Still higher flow rates resulted in significantly lower changes in SPR responses, to about half of its maximal value.

To validate more closely the carbohydrate-specific component in the *E. coli* interaction at different flow rates, we used a Au-graphene-BSA interface without modification, as well as the *E. coli* strain UTI89 Q133K that expresses a (Glu133 to lysine) mutant FimH that proves totally dysfunctional in terms of mannose binding [24,36,48]. Blocking the surface with BSA eliminated all shear force-dependent surface adsorption of wild type UTI89, with a similar SPR signal at all flow rates at a background level of ≈ 200 pixels (Figure 2). The mutant UTI89 Q133K showed some remaining shear force-dependent behavior similar to that of wild-type UTI89 on Au-graphene-HM, despite coating with BSA against non-mannose-directed protein binding (Figure 3a, Group 3). However, in the presence of a specific FimH-carbohydrate interaction, the shear-enhanced adhesion became much more pronounced, which clearly points out the importance of mannosidic substrates on the binding capability of *E. coli* strains under flow. Having established this concept, one may now start to distinguish molecular properties of FimH variants that allosterically regulate adhesion behavior under shear stress from those permitting the binding of mannose.

### 3.4. E. coli Strains UTI89 and K12 Display the Strongest Shear Force-Enhanced Affinity

The adhesion behavior of *E. coli* clinical isolates from human patients was investigated to gain a better understanding of pathogen interactions at the initial stage of infection. The *fimH* genes of the newly isolated *E. coli* strains from osteoarticular infections were sequenced and compared to the amino acid sequence of the laboratory *E. coli* strain K12 MG1655 (Table 1). Together with AIEC strains associated with Crohn’s disease [32], the *fimH* sequences form a complete panel of amino acid variants found naturally in the FimH adhesin from in total 14 *E. coli* clinical isolates, among which one uropathogenic *E. coli*, five from osteoarticular infections, one fecal isolate and seven Crohn’s disease-associated strains (two fecal and five from biopsies) (Table 1).

The results summarized in Figure 3 are the SPR intensities upon interaction of the different *E. coli* strains with Au-graphene-HM as a function of flow rate. They confirm that a minimal shear (>5 µL·min^−1^) is needed to observe increased adhesion, as evidently, the flow rate is known to increase the adhesion rates in microfluidic devices [49]. Remarkably, for the AIEC strains, the optimal flow rate to achieve maximal bacterial interaction with the glycosylated surface is consistently lower (10 or 20 µL·min^−1^) than for the other disease-associated *E. coli* strains, where higher flow rates (up to 30 µL·min^−1^) result in further increased bacterial interaction with the HM-modified SPR interface (Figure 3a). SPR signals for all strains on a Au-graphene-HG surface were observed near the background level of 200 pixels and did not vary as a function of the flow rate (Figure 3b).

### 3.5. The AIEC Strain LF82 Displays the Highest Affinity under Static Conditions

The SPR experiments demonstrate the potential to study the dynamic binding of *E. coli* to mannosylated interfaces and probably approaches much better what happens just prior to infection of epithelial surfaces. However, for practical reasons, bacterial adhesion tests are more frequently performed under static conditions, which resembles more closely the bacterial behavior in, for example, biofilms. It is also useful to be able to compare bacterial adhesion under flow with static conditions because of well-established protocols and techniques for the latter. *E. coli* can keep guinea pig erythrocytes in suspension by using its fimbriae to form cross-links between its FimH adhesin and mannosides in the glycocalyx of the red blood cells. This process of hemagglutination and its specific inhibition using α-d-mannosides is indicative of type-1 fimbrial expression on the bacterial surface [50].

**Figure 4:**
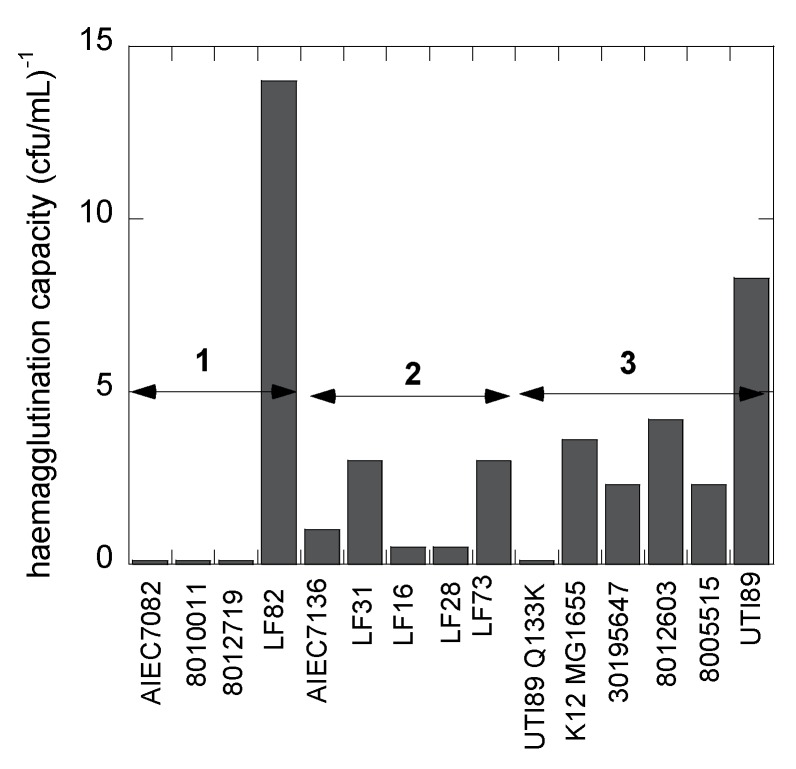
The capacity (taken as the inverse of the titer) of the *E. coli* strains to hemagglutinate guinea pig red blood cells under static conditions measures mannose-sensitive recognition by type-1 fimbriae. The groups classified as shear-dependent bacterial adhesion are indicated as in Table 1 and Figure 3. All bacterial hemagglutination were susceptible to inhibition by FimH antagonists [52].

The ability of the 14 *E. coli* strains to adhere under static conditions was evaluated using agglutination assays (Figure 4). The absence of agglutination of guinea pig red blood cells at the highest bacterial concentration (typically 0.6 × 10^10^ cfu·mL^−1^) indicates the absence of mannose-sensitive type-1 fimbriae on the bacterial surface. This is the case for two *E. coli* isolates from osteoarticular infections and one from the feces of a Crohn’s disease patient (Table 1). All hemagglutinating strains could be specifically inhibited by micromolar concentrations of heptyl α-d-mannoside [6] and several other mannosides [51,52]. The LF82 AIEC strain exhibits the highest hemagglutination capacity, congruent with it being an excellent colonizer of intestinal cells expressing high levels of the CEACAM6 mannosylated receptor protein [53].

### 3.6. Human E. coli Pathovars Display a Different Optimum Flow Rate for Enhanced Adhesion

Analysis of the SPR data allows the differentiation of three groups of FimH-mediated *E. coli* adhesion behaviors under shear flow (Figure 3). The first four strains, classified as Group 1 (Table 1), were characterized as being *quasi* indifferent to flow rate. These *E. coli* strains, except for *E. coli* LF82, showed no hemagglutination, indicating a lack of *fimH* expression. Group 1 *E. coli* only bind at a low SPR level (<500 pixels) that does not vary much with the flow rate, although a light shear force-dependent binding pattern is visible (Figure 3a). Such interactions may be indicative of remaining protein interaction events of the adhesins with the surface, independent of mannose recognition, especially because three of the strains do not hemagglutinate guinea pig red blood cells. The UTI89 Q133K mutant, that is incapable of binding mannose, also demonstrates such a pattern, whereas the purified FimH lectin domain does not (Figure 3a, Group 3). Group 2 comprises five different *E. coli* strains, all of which belong to the AIEC type and are isolated from patients with ileal Crohn’s disease (Table 1). They all show shear force-enhanced affinity at 10–20 µL·min^−1^ flow rate and exhibit a moderate agglutination capacity (Figure 4). Group 3 *E. coli*, including uropathogenic UTI89, the K12 MG1655 fecal strain and *E. coli* isolated from osteoarticular infections, show clearly enhanced interaction with shear, with a maximum adhesion occurring at 30 µL·min^−1^. It becomes evident from these findings that there is no direct correlation between shear-forceinduced adhesion and hemagglutination capacity (Table 1).

### 3.7. Amino Acid Variations in FimH from AIEC Strains Moderate Shear Force Regulation

**Figure 5:**
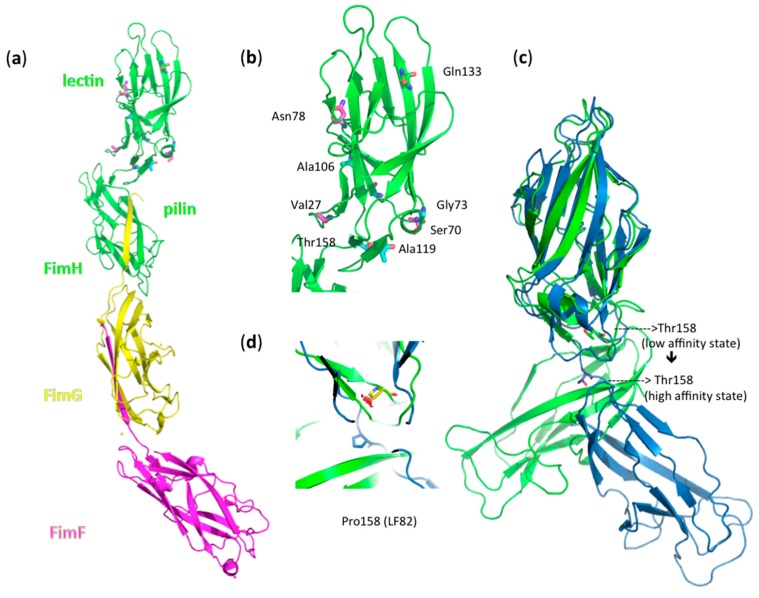
(**a**) The fimbrial tip of *E. coli* K12 type-1 fimbriae, which represents FimH in its low-affinity state [4]: a FimH TDA with lectin and pilin domain-FimG-FimF macromolecular assembly (PDB Entry 3JWN) [4]. Amino acid variations in FimH between the 14 *E. coli* strains (Table 1) are indicated in ball-and-stick models. (**b**) Zoom out on the fimbrial tip-attached FimH lectin domain. Variants of the Ala27, Ser70 and Asn78 residues, which typify a separate AIEC clade [31], have been indicated in magenta; the Gln133Lys (Q133K) mutation, introduced in FimH to disable mannose binding, is shown in yellow; other variations are in cyan. (**c**) Superposition onto the high-affinity conformation of FimH (blue; PDB Entry 1KLF) [8] illustrates a large shift of amino acid position 158 in the linker of the FimH TDA. (**d**) The change of Thr158 to proline (yellow) in LF82 FimH gives steric clashes (red clouds) in the low-affinity FimH conformer (green), but not in the high-affinity FimH state (blue).

Most striking is the difference in behavior of the Crohn’s disease-associated AIEC strain LF82 and the uropathogenic strain *E. coli* UTI89. The LF82 strain exhibits strong hemagglutination, but remains indifferent to shear. UTI89 also has a high agglutination capacity, accompanied by a strong dependence on shear force for the regulation of the affinity of FimH. The Thr158Pro mutation is the single amino acid distinguishing these strains (Table 1). Superposition of the crystal structures of K12 FimH in its low-affinity state (Figure 5a,b) onto the high-affinity state of the TDA FimH indicates linker chain extension and a large shift moving this residue into the flexible linker region between pilin and the lectin domain of FimH for the high-affinity, extended conformer (Figure 5c). Mutation of Thr158 to proline and geometrical minimization in the low-affinity FimH conformer leads unavoidably to steric clashes, but not in the high-affinity FimH conformation (Figure 5d). This indicates that the Thr158Pro FimH variant of *E. coli* LF82 may have its two domains already separated by an extended linker, keeping the FimH lectin in the high-affinity state, which is congruent with the good binding of LF82 under static conditions (Figure 4). The two domains already straightened up around the hinge would result in a decreased shear-force enhancement of the affinity of these FimH adhesins (Figure 3 and Table 1). Our results are in agreement with the reported invalidation of shear-force dependence by the Val156Pro mutation of FimH from the F18 fecal *E. coli* strain [17]. The F18 *E. coli* strain requires shear-force enhancement of affinity, even to bind the high-affinity receptor mannotriose, but the Val156 to proline mutation renders its binding independent of shear-force, even for the low-affinity monomannose ligand [54].

## 4. Discussion

Naturally occurring variations in FimH from *E. coli* pathotypes are known to influence bacterial adhesion under shear flow, even though the residues making up their mannose-binding pocket remain highly conserved [55]. The first observations of shear-force enhanced adhesion were made with the fecal *E. coli* F18 strain that did not hemagglutinate red blood cells under static conditions [10]. Shear force enhancement has not been further investigated using *E. coli* isolates displaying a natural variation in FimH amino acids, which potentially plays a role in the allosteric regulation of receptor affinity for different *E. coli* pathotypes, until very recently [15]. We have undertaken SPR experiments using clinical clones that display variable amino acids of FimH, including novel *E. coli* isolates from osteoarticular infections. The adhesion behaviors of in total 14 clinically relevant *E. coli* clones have here been examined under flow and under static conditions.

Different adhesion behaviors of the bacterial strains can also be related to their particular presentation on the bacterial cell envelope, decorated with lipopolysaccharides, flagella and fimbriae. The biosynthesis of type-1 fimbriae varies under different growth conditions and even as a consequence of single allelic variations in the *fimH* gene [1]. Type-1 fimbrial expression is strongly phase-regulated and has been positively selected for in pathogenic *E. coli* to enable rapid adaptation to newly invaded environments [50]. The mannose-sensitive hemagglutination by the strains allows a verification of the presence of type-1 fimbrial adhesins. For example, *E. coli* strains LF28 and 8005515 have identical FimH sequences (Table 1) and are both characterized by an adhesion pattern under flow that is strongly influenced by shear force (Figure 3a). However, they adhere optimally under slightly different flow rates and therefore must be considered in different categories, Groups 2 and 3, respectively. The difference in adhesion behavior could be related to the lower capacity of *E. coli* LF28 compared to *E. coli* 8005515 to haemagglutinate guinea pig red blood cells under static conditions (Figure 4).

Another mechanism that contributes dominantly to type-1 fimbriae-mediated bacterial adhesion is the amino acid composition of the fimbrial shaft, with FimA as the major structural component and FimF and FimG as minor pilins. Inter-strain sequence variation in *E. coli fimA* is more significant than in *fimH* genes [1] and, thus, has to be taken into account when studying the adhesion behavior of clinically-relevant *E. coli* clones. The amino acid sequences for the FimA and FimG mature pilin proteins are identical in the *E. coli* LF82 and UTI89 isolates, the two strains with the most different reactions to flow rate (Figure 3a), and only in FimF, a single amino acid change, Ala35Val, can be noted. Combined with the observation that both strains also have a good mannose-sensitive hemagglutination capacity (Figure 4), the optimal flow rate for shear force-enhanced adhesion is more probably determined by amino acid sequence variation in the FimH adhesin of the type-1 fimbrial appendages of *E. coli*. We have projected those amino acid variations onto the crystal structures of full-length FimH proteins (Figure 5).

It is now generally believed crucial for uropathogenic *E. coli* to be able to withstand shear forces. The biomechanical bases of shear force enhancement are structural changes taking place in the FimH TDA upon the formation of catch bonds with mannose. When FimH adopts a stretched, elongated conformation in the tip of the fimbriae, this also corresponds to increased mannose binding affinity [5,14,15,56]. *E. coli* UTI89, like *E. coli* K12, is able to undergo fimbrial extension under the influence of shear stress, because FimH is able to undergo the conformational changes that transduce to the remainder of the type-1 fimbriae [57]. The results described here show that the affinity of Crohn’s-associated AIEC strains to aminoheptyl α-d-mannoside-modified graphene on a gold plasmonic surface (Au-graphene-HM) is less enhanced under shear force than for the other tested *E. coli* strains, and the maximal enhancement is already attained at a lower flow rate. The AIEC strain LF82 demonstrates this at the extreme, by displaying a *quasi*-shear force-independent adhesion with an already optimal affinity under static conditions. The high-affinity state of FimH has been associated with the elongated conformation of the fimbrial tip TDA (Figure 5c), where the inter-domain linker is extended and separates the two domains [15]. Although hard structural information is lacking for the moment, the Thr158Pro mutation in LF82 FimH could preclude the compressed, low-affinity conformation of FimH. This state of the FimH adhesin is somehow comparable to the structure of the invasive tip complex from Afa/Dr fibrils, as determined using NMR [57].

**Figure 6:**
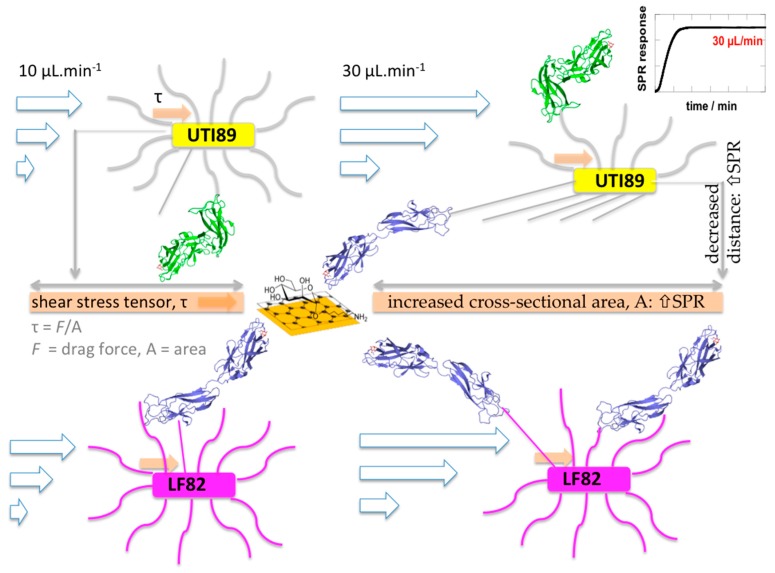
Uropathogenic *E. coli* UTI89 relieved from shear stress through fimbrial spring-like behavior, initiated by opening of the FimH TDA around the hinge and domain extension when forming catch bonds with α-d-mannoside receptors on the graphene interface. Because of fimbrial stretching, bacterial adhesion can take place over a larger area, leading to a decrease in shear stress under fluid flow. At the same time, the bacterium is pulled towards the surface, and the distance to the surface decreases [17,40,56]. Both factors increase interaction events taking place close to the surface and may explain the more intense SPR signals. On the other hand, the FimH TDA from AIEC LF82 is unable to undergo the conformational changes that initiate type-1 fimbrial stretching.

In the AIEC pathovars, the presence of mutations in the inter-domain region of the FimH lectin domain (Table 1, Figure 5) may thus hinder or prevent the change towards the low-affinity conformation of FimH and disable the FimH adhesin to act as an initiator of type-1 fimbrial rod extension (Figure 6). The elastic properties of the type-1 fimbriae can have a dramatic effect on the lifetime of the receptor bond. Simulations have demonstrated that a bond linked to a rigid rod tends to break at high forces and survives for relatively short times, whereas a bond linked to a fully-extensible rod tends to break at (too) low forces and survives much longer times at higher forces [58]. The accumulation of the bacteria onto the interface is aided by an increased lifetime of the bonds made by the fimbrial appendages that function as extensible rods when the bacterium is dragged over the surface by shear forces [54]. The bacteria arrest from rolling over and sticking to mannosylated surfaces by making catch bonds by FimH on type-1-fimbriae. The consequent decrease in the distance of the pathogen to the surface, together with an increase in the number of binding events happening close to the plasmonic surface, correlates with an increased SPR intensity (Figure 3a and Figure 6). Interesting in this regard is that the FimA major pilin of the type-1 fimbrial shaft is identical in sequence between the UTI89 and LF82 *E. coli* strains. Differences in FimA can therefore not be used to explain the large difference in SPR responses of these two strains.

FimH variants that cannot interconvert between the two conformational states are attenuated during chronic bladder infection [59]. Here, we revealed that the FimH variant from LF82, a prototypical AIEC strain for Crohn’s disease, can display exactly this inconvertible conformation phenotype. LF82 FimH is hampered from switching between conformations because of the Thr158 to proline mutation. The potential of the FimH adhesin to switch its conformation back from the high- to the low-affinity state has indeed been associated with the potential to shed antibodies bound to it and, consequently, for *E. coli* to persist in colonization [60,61]. Amino acid variations in the inter-immunoglobulin-like domain region of the FimH adhesin are epitopes for antibody recognition and immune response regulation. In the case of LF82 FimH, keeping the FimH adhesin in the elongated, high-affinity state and the hinge domain open for antibody recognition could be related to the exacerbated inflammation typical of Crohn’s disease [60].

The FimH adhesin has affinities for mannotriose and mannopentaose (K_d_ = 20 nM) that are 100-fold higher than for α-d-mannose (K_d_ = 2 μM) [62,63]. A high dependence on shear force-enhanced adhesion makes perfect sense in regard to monomannose being the low-affinity ligand targeted by *E. coli* in the urinary tract [8]. Strangely enough, even on surfaces coated with mannotriose, increasing numbers of stationary bacteria have been observed under flow conditions [54], however without a clear shear force-enhanced adhesion. The latter scenario may correspond to less molecular interactions happening close enough to the plasmonic surface to contribute to SPR responses, congruent with our observations for the strongly hemagglutinating *E. coli* LF82 (Figure 6).

## 5. Conclusions

Au-graphene interfaces modified with high-affinity ligands for FimH have here shown potential as plasmonic surfaces to obtain information on laminar flow-enhanced interactions of *E. coli* clinical isolates. The interfaces could easily be modified with aminoheptyl α-d-mannopyranoside, a ligand with an affinity (K_d_ = 20 nM) just like mannotriose, to study specific binding of *E. coli* under flow. The low influence of shear stress on the adhesion capacity of AIEC strains may indicate that in the gut, enteric *E. coli* have the opportunity to bind glycan receptors with a structure based on the mannotriosidic core of natural oligomannosides [64], rather than being restricted to binding a single mannose at the non-reducing end of *N*-linked glycans, as is known in the bladder [8]. Furthermore, graphene is a much more protein-friendly material than, for example, gold. It allows hydrophobic encounters to take place and gives a basal level of interaction similar to biological surfaces. The technique developed here for studying bacterial-host glycan interactions appears more biologically compatible with bacterial adhesion in the human gut than models based on monomannose on gold surfaces.

The presence of mannotriosidic receptors for FimH may have eliminated the need for enteric *E. coli*, such as AIEC, to enhance affinity through a shear-dependent mechanism. It remains hitherto unknown whether any of the other mutations found in the inter-domain region of the FimH adhesins of Crohn’s-associated AIEC strains [32] (Figure 5, Table 1) would favor their persistence in chronic inflammatory bowel diseases by a similar mechanism. However, we have shown that AIEC bacteria detach more readily under increased flow rates than other *E. coli* pathovars. The reduced capacity of AIEC strains to relieve shear stress and prevent bacterial detachment may be well due to the different physico-mechanical (shear) and chemical (glycan receptor) conditions imposed on them during the colonization of epithelial linings. AIEC colonize the thick, mucosal layers of the intestinal epithelium, whereas uropathogenic *E. coli* bind the highly glycosylated urothelial cells that undergo large shear during voiding and filling of the bladder. A lack of dynamism in response to shear may also render AIEC more susceptible to soluble inhibitors, such as anti-adhesives that counteract the FimH adhesin [65]. In conclusion, our results add valuable insights into the way bacteria colonize specifically glycosylated surfaces under fluid flow.

## Figures and Tables

**Table 1:** *E. coli* clinical isolates and the mutant (UTI89 Q133K) used in this work: sequences (mannose-binding residues are shown in green, variant residues in cyan, except the ASN clade of AIEC fecal strains [32] with residues Ala27, Ser70 and Asn78 in magenta), origin, agglutination titer and group. Group categorization is according to the flow rate optimal for adhesion (1 = no optimum flow rate; 2 = optimal adhesion at 10 or 20 μ·mL^−1^; 3 = maximum SPR intensity at 30 μ·mL^−1^ and exceeding 2000 pixels; neg. = negative).

*E. coli* Strain	*fimH* Sequence Amino Acid Differences with *E. coli* K12 MG1655	Origin of *E. coli* Strain	Agglutination Titer (× s10^9^ cfu·mL^−1^)	Group
***10*** ***20*** ***30*** ***40*** ***50*** ***60***				
FACKTANGTA IPIGGGSANV YVNLAPVVNV GQNLVVDLST QIFCHNDYPE TITDYVTLQR				
***70*** ***80*** ***90*** ***100*** ***110*** ***120***				
GSAYGGVLSN FSGTVKYSGS SYPFPTTSET PRVVYNSRTD KPWPVALYLT PVSSAGGVAI				
***130*** ***140*** ***150*** ***158***				
KAGSLIAVLI LRQTNNYNSD DFQFVWNIYA NNDVVVPT **K12 MG1655 lectin domain**				
AIEC7082	ASN Thr158Ala	Crohn’s feces	neg.	1
8010011	ASN	knee prosthesis	neg.	1
8012719	ASN	hip prosthesis	neg.	1
LF82	ASN Thr158Pro	Crohn’s biopsy	0.07	1
AIEC7136	ASN Gly73Arg	Crohn’s feces	0.97	2
LF31	ASN Gly66Ala Ala106Thr	Crohn’s biopsy	0.30	2
LF16	ASN Gly73Trp	Crohn’s biopsy	2.01	2
LF28	A Ala119Val	Crohn’s biopsy	1.91	2
LF73	ASN	Crohn’s biopsy	0.32	2
UTI89 Q133K	ASN Gln133Lys	uropathogenic mutant FimH	neg.	3
K12 MG1655		fecal	0.28	3
30195647		fecal	0.43	3
8012603		total hip replacement	0.24	3
8005515	A Ala119Val	incomplete hip replacement	0.42	3
UTI89	ASN	uropathogenic	0.12	3

## Supplementary Materials

The following are available online at www.mdpi.com/2079-7737/5/1/14/s1, Figure S1: Formation of Au-graphene. Table S1: Characterization of (a) carbohydrate-modified Au-graphene interfaces and (b) Au substrates. Figure S2: SPR sensorgrams as a function of flow rate for *E. coli* UTI (10^8^ cfu·mL^−1^) to Au-graphene-HM interfaces.

## Abbreviations

The following abbreviations are used in this manuscript:

AIEC	adherent-invasive *Escherichia coli*
BSA	bovine serum albumin
HG	aminoheptyl α-d-glucopyranoside
HM	aminoheptyl α-d-mannopyranoside
OD	optical density
PBS	phosphate-buffered saline
SPR	surface plasmon resonance
TDA	two-domain adhesin
