# Supplementary material for: Differentiation of Crohn’s Disease-Associated Isolates from Other Pathogenic Escherichia coli by Fimbrial Adhesion under Shear Force
Source: Biology (Basel). 2016 Apr 1;5(2):14. doi: 10.3390/biology5020014 (PMC4929528; doi:10.3390/biology5020014)
Supplement: Supplementary file 1 [file biology-05-00014-s001.pdf]

Supplementary Materials: Differentiation of Crohn's Disease-Associated Isolates from Other Pathogenic *Escherichia coli* by Fimbrial Adhesion under Shear Force

Sabine Szunerits, Oleksandr Zagorodko, Virginie Cogez, Tetiana Dumych, Thibaut Chalopin, Dimitri Alvarez Dorta, Adeline Sivignon, Nicolas Barnich, Anne Harduin-Lepers, Iban Larroulet, Aritz Yanguas Serrano, Aloysius Siriwardena, Amaia Pesquera, Amaia Zurutuza, Sébastien G. Gouin, Rabah Boukherroub and Julie Bouckaert

Fabrication of Au-graphene (graphene@Au) SPR Interfaces

The prisms used for SPR assays have a refractive index n = 1.569 (HBAK1, Schott) and have been modified by a Ti adhesion layer of 2 ± 0.5 nm and a Au thin film of 47 ± 2 nm deposited both by sputtering under vacuum. Graphene was synthesized as reported before [1], on polycrystalline Cu foils (25-μm-thick Alfa-Aesar, purity 99.98%) in a cold walled chemical vapor deposition (CVD) reactor, namely the Cu foils were annealed at 1000 °C under the flow of H_2 and Ar prior to graphene growth. Then, CH_4 was introduced in the chamber to carry out the graphene growth. Finally, the sample was cooled under Ar atmosphere. Graphene was transferred onto the gold SPR interfaces by a modified wet chemical transfer process (Figure S1A) [1]. Briefly, polymethylmethacrylate (PMMA, Microchem 495k) was spin-coated onto the graphene-coated Cu foil. The PMMA/graphene/Cu sample was baked at 150 °C for 2 min and then slowly cooled to room temperature. Backside graphene was removed by oxygen plasma treatment. Copper was dissolved using a commercial copper etchant solution (Transene). Graphene/PMMA was rinsed with de-ionized water 5–6 times to remove any ion contamination. Finally, the graphene was transferred onto the SPR substrate. After 10 min (for natural removal of water underneath the graphene), another slow backing step at 90 °C was carried out to remove the trapped water and to increase the contact between graphene and the substrate. Subsequently the PMMA layer was removed by dipping the sample in acetone for 30 min. The SPR substrate with the transferred graphene was then rinsed with isopropanol and dried by mild nitrogen blow.

Micro-Raman spectroscopy measurements were performed on a Horiba Jobin Yvon LabRam HR micro-Raman system combined with a 473-nm (1 mW) laser diode as excitation source. Visible light is focused by a 100× objective. The scattered light is collected by the same objective in backscattering configuration, dispersed by a 1800 mm focal length monochromator and detected by a CCD camera. The typical Raman 2D band at ≈ 2710 cm^{-1} and the G band at ≈ 1580 cm^{-1} of graphene are observed (Figure S1B) with only small defects (D band at 1359 cm^{-1}), indicating the high quality of graphene@Au interfaces. Evaluation of the chemical nature of the graphene film by XPS revealed the presence of a dominant peak at 284.7 eV assigned to the sp^2 graphene framework along with smaller peaks at 285.1 eV (sp^3 type carbon, C-H) and 286.2 eV (C-O impurities) (Figure S1C).

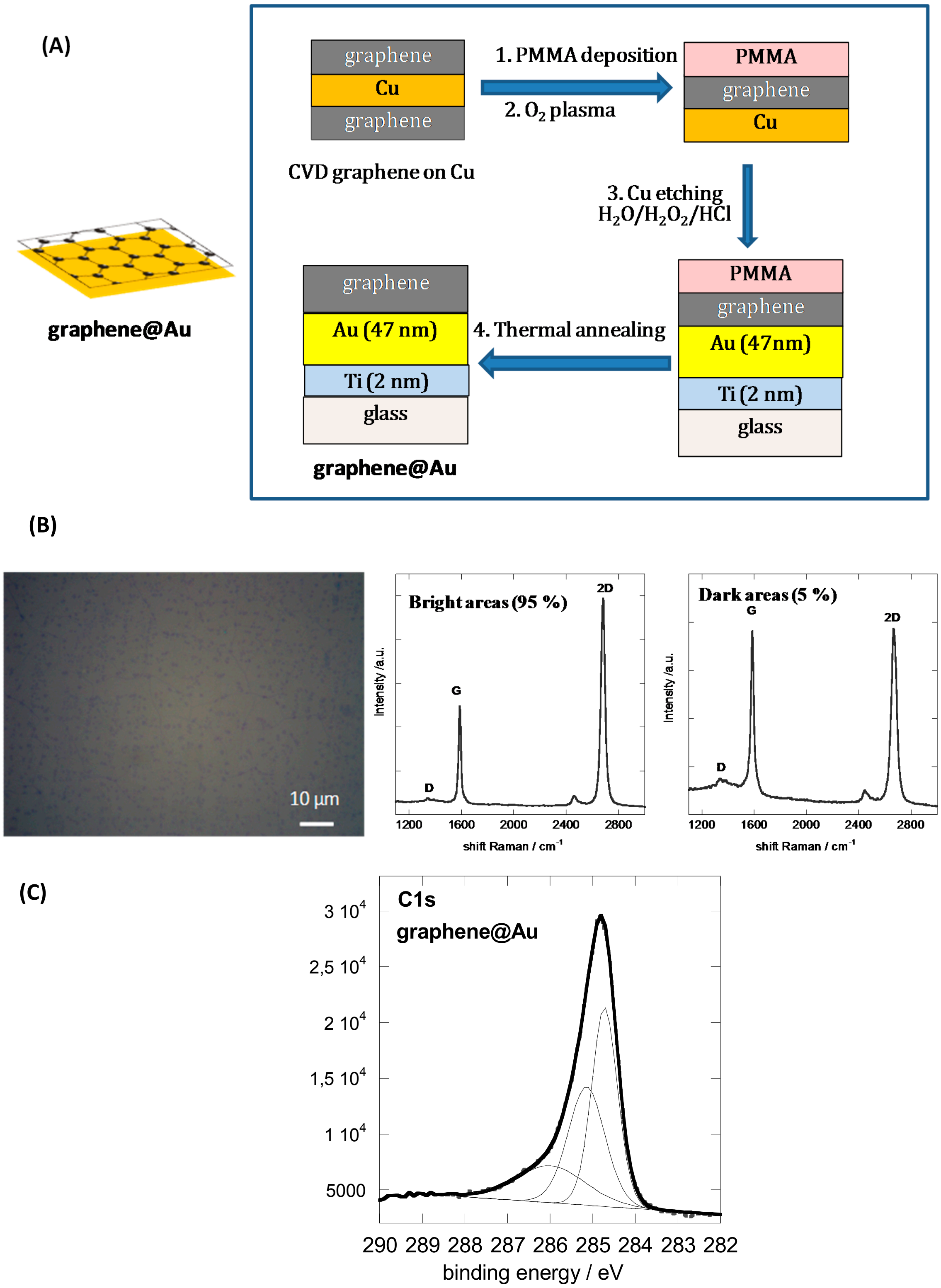

Figure S1. (**A**) Formation of Au-graphene interfaces. (**B**) SEM (left) and Raman (right); (**C**) C_{1s} high-resolution XPS spectrum of a graphene@Au substrate.

XPS experiments were performed using a PHI 5000 VersaProbe—Scanning ESCA Microprobe (ULVAC-PHI, Kanagawa, Japan) instrument at a base pressure below 5×10^{-9} mbar. Monochromatic AlK_{α} radiation was used and the X-ray beam, focused to a diameter of 100 µm, was scanned on a 250 µm × 250 µm surface, at an operating power of 25 W (15 kV). Photoelectron survey spectra were acquired using a hemispherical analyzer at pass energy of 117.4 eV with a 0.4 eV energy step. Core-level spectra were acquired at pass energy of 23.5 eV with a 0.1 eV energy step. All spectra were

acquired at 90° between X-ray source and analyzer and with the use of low energy electrons and low energy argon ions for charge neutralization. After subtraction of the Shirley-type background, the core-level spectra were decomposed into their components with mixed Gaussian-Lorentzian (30:70) shape lines using the CasaXPS software. Quantification calculations were performed using sensitivity factors supplied by PHI.

The amount of glycan loaded on the graphene@Au interface was analyzed by treatment with a phenol/H_2SO_4 solution as described previously [2].

Table S1. Characterization of carbohydrate-modified Au-graphene (a) and Au (b) substrates: atomic percentage (at. %) of elements as determined by XPS, and sugar loading of (**a**) modified Au-graphene and (**b**) Au interfaces determined by the Molisch's test [2].

Glycans	C1s at. %	O1s at. %	N1s at. %	Sugar Loading molecules·cm^{-2}
**(a) Au-graphene**				
aminoheptyl α-D-mannoside (**HM**)	72.59	25.34	2.07	7.1 ± 0.6 × 10^{12}
aminoheptyl α-D-glucoside (**HG**)	72.97	25.04	2.09	6.9 ± 1.2 × 10^{12}
nitrophenyl α-D-mannoside (**3**)	80.51	17.10	2.39	7.6 ± 0.6 × 10^{12}
nitrophenyl α-D-glucoside (**4**)	77.23	20.54	2.23	7.4 ± 0.4 × 10^{12}
nitrophenyl β-D-glucoside (**5**)	79.56	18.11	2.33	7.5 ± 0.5 × 10^{12}
D-mannose (**1**)	73.48	26.52	-	5.8 ± 0.6 × 10^{12}
methyl α-D-mannoside (**2**)	75.23	24.77	-	5.4 ± 0.7 × 10^{12}
**(b) Au**				
aminoheptyl α-D-mannoside (**HM**)	71.01	26.52	2.47	8.5 ± 0.6×10^{12}

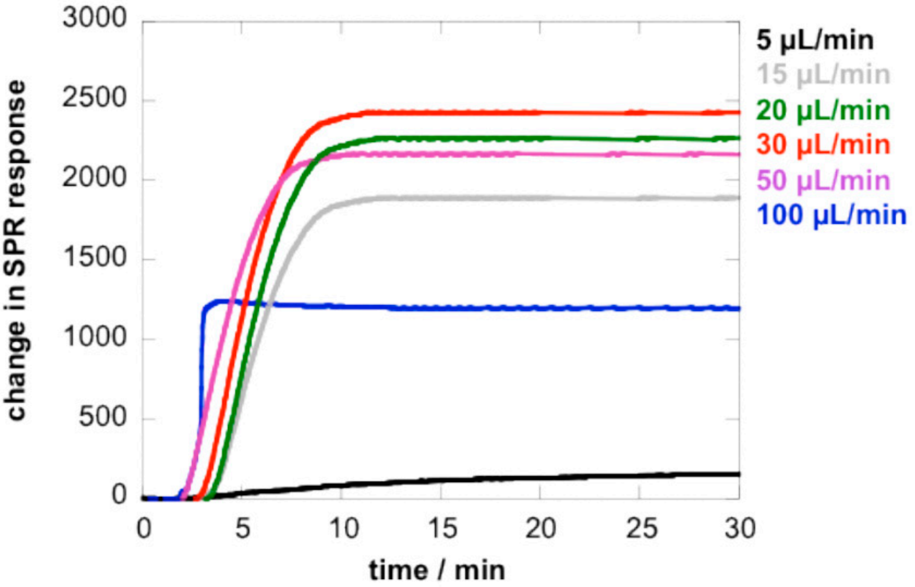

Figure S2. SPR Sensograms as a function of flow rate for E. coli UTI (10^8 cfu·mL^{-1}) to Au-graphene-HM interfaces.

References

1. Li, X.; Cai, W.; An, J.; Kim, S.; Nah, J.; Yang, D.; Piner, R.; Velamakanni, A.; Jung, I.; Tutuc, E. Large-area synthesis of high-quality and uniform graphene films on copper foils. *Science* **2009**, *324*, 1312–1314.
2. Barras, A.; Martin, F.A.; Bande, O.; Baumann, J.S.; Ghigo, J.M.; Boukherroub, R.; Beloin, C.; Siriwardena, A.; Szunerits, S. Glycan-functionalized diamond nanoparticles as potent *E. coli* anti-adhesives. *Nanoscale* **2013**, *5*, 2307–2316.
